# How humour travels in the new and dynamic mediascape: a case study of a short video platform, Little Red Book, and an online teaching platform, Rain Classroom

**DOI:** 10.1057/s41599-023-01822-8

**Published:** 2023-06-12

**Authors:** Lisi Liang

**Affiliations:** Sun Yat-Sen University, No. 135, Xingang Xi Road, Guangzhou, P. R. China

**Keywords:** Language and linguistics, Cultural and media studies

## Abstract

As a vital part of translation studies, humour has drawn scholarly attention for decades, with classifications that range from Zabalbeascoa’s (The Translator 2(2):235–257, 1996) six types of jokes to Chiaro and Piferi’s (It’s green! It’s cool! It’s Shrek! Italian children, laughter and subtitles. In: Di Giovanni E, Elefante C, Pederzoli R (eds) Écrire Et Traduire Pour Les Enfants—writing and translating for children. Peter Lang, Brussels, 2010, p. 285) “Verbally Expressed Humour”. However, they are mainly related to printed pages, theatre, and film. Little research touches on the new media, which significantly impacts how information is produced and disseminated and how consumers react to and engage with these trendy platforms (Díaz-Cintas, Remael. Audiovisual translation: subtitling. Routledge, London and New York, 2021, p. 1). This significant gap in the video-sharing platforms on humour translation is the focus of this paper which intends to fill. This paper explores how humour is created and reconstructed in the dominant and constantly evolving new media era. Driven by the niche of an interdisciplinary study concerning humour and creative subtitles, the present research conducts a linguistic and semiotic analysis of humorous discourses and emojis in the Chinese contexts of the short video platform Little Red Book and the online teaching platform Rain Classroom. As the study implies, humour can be strengthened through diverse semiotic possibilities to provide better viewing experiences that bring about entertaining and educational outcomes.

## Introduction

Since 2000, modern viewership has evolved from traditional “passive media spectatorship” to “participatory culture” (Jenkins, 2006, p. 3). Contemporary audiences are “emotionally invested” (Orrego-Carmona, 2018, p. 322) in media products immersing themselves as “active members of global collectivities” (Pérez-González, 2013, p. 158). This paper demonstrates such emotional evolution of the consumption and production of video-sharing and educational platforms in the digital era. The conventionally isolated division between dubbing and subtitling practices seems insufficient to account for today’s dynamic and intricate mediascape in which “a profusion of titling elements” are used to blend in with the audiovisual surroundings (Pérez-González, 2007, p. 67). Under these contexts, subtitles with innovation emerged to address the global and digital viewership characterised by mobilisation, gamification, customisation, ubiquity, immediacy and social integration (Pérez-González, 2007, p. 67; Fernández-Costales, 2018, pp. 297–311; Orrego-Carmona, 2018, p. 323). These representative instantiations can be found over the past two decades through Nornes’s (1999) *abusive subtitling*, McClarty’s (2014) *creative subtitles*, Chaume’s (2018) three different types of fun AVT techniques: *fundubs, fansubs and funads*, Yang’s (2020) *danmuku subtitling*, Lee’s (2021) *non-representational subtitling*, etc. These innovative subtitles give end users a high level of creativity and meditation in creating translation and its corresponding special effects. Although studies of multilingual scripts and multimodal resources in dubbing and subtitling for the past two decades have been available (Díaz-Cintas and Remael, 2007; Pérez-González, 2013), publications on the innovative subtitles embedded in contemporary social media featuring cross-disciplinary natures between interlingual, intralingual and multimodal translation practice remain scant. This paper aims to justify the role of transcreated subtitles in extending the boundary of translation studies to interdisciplinary fields between social semiotics and translation studies, with particular references to humour translation. It specifically relates to its individuality and creativity around multimodal translated vlogs circulated through video-sharing platforms, the Little Red Book (original Chinese name as “小红书”, hereafter, Red) and the educational platform Rain Classroom (original Chinese name as “雨课堂”, hereafter, Rain), proving that non-professional subtitlers act as a significant, visible player in the transmedia era.

## Why Little Red Book and Rain Classroom?

The selection of the short video platform Little Red Book and the educational platform Rain Classroom as case-study instances are based on twofold benefits and implications associated with the humour embedded from the two different types of platforms in the digital mediascape. On the one hand, the essence of short-video platforms carries user-generated content, a form of customer co-creation value (Xie et al., 2019), tapping into the amateur-led grassroots participation in the digital age that lies at the heart of this paper. Red is worth investigating, yet it needs more academic attention despite gaining popularity among the younger generation in China (Liang, 2023). On the other hand, as opposed to Citgez et al.’s (2021) statement that more popular social platforms seem to contain a less educational function, our study argues that social platforms may carry valid pedagogical values thanks to danmu technology. This will be supported shortly in our case-analysis section by the key educational platform Rain Classroom, designed and developed by Tsinghua University in 2016. According to Yu and Yu (2022, p. 1215), Rain Classroom proves to have higher learning attainment compared to traditional learning based on eight attributes: “effectiveness, efficiency, satisfaction, learnability, memorability (opportunities to learn from) errors, cognitive load and timeliness” throughout pre-class, in-class and after-class experiences. Regardless of the popularity and usabilities Rain Classroom received in education, there is limited rationale for its reception from students’ perspectives, in which the humorous effect may facilitate student learning initiatives. This paper aims to explore these new and humorous effects and pedagogical functions drawn from the social media platform Little Red Book and the educational platform Rain Classroom.

## Research questions

Since our study distinguishes itself through its educational and pedagogical nature in collaboration with danmu comments and humour translation in the teaching contexts, the study aims to answer three research questions:

1. What characterises humour in the Chinese social media Little Red Book and online teaching platform Rain Classroom?
2. How to translate such humour in the Chinese social media Little Red Book and online teaching platform Rain Classroom?
3. What potential practical, methodological and theoretical contributions does humour translation bring to translation studies?

Based on the interactions with previously existing literature, the following sections conceptualise a theoretical frame that structures our case study of comprehending humour drawn from short-video sharing and educational platforms.

## Conceptual frame of approaching humour in the context of social media platforms

In terms of theories, based on the theory of polysystem (Even-Zohar, 1979) and multimodality (Baldry and Thibault, 2006), this article will position itself on the confluence of research on humour and subtitling studies. Polysystem is selected based on the heterogeneous and dynamic sub-systems one has to consider when comprehending humour because comprehension of humour can be improved by taking into account the various and dynamic sub-systems that constitute it. This is similar to subtitling practices in the multimedia and constantly evolving audiovisual 5G age. Novel and multimodal semiotic systems, such as real-time danmu/danmaku, user-oriented gift-giving, and emojis instead of written words/characters in messages, can enhance the understanding of humour (Liang, 2023, p. 2). Kress’s (2004, pp. 116–119) approach, which looks at the interaction of “writing, layout, colour, speech, music and image”, is valuable for analysing how media platforms collaborate to reshape humour translation.

Methodologically, it will adapt Gottileb’s (1997, p. 143) four semiotic channels to transmit information in feature films or television programmes into three, namely verbal channel, visual channel, and the combination of these two to group examples fit explicitly in the two cases, the social and short video platform, Little Red Book and the educational platform, Rain Classroom. I argue that Gottileb’s two semiotic channels—non-verbal audio and non-verbal visual—are challenging to identify without fully considering the context. It is more likely to identify examples containing humorous effects simultaneously considering contextual audio and visual elements because of the acceptability of the “context of situation” (Malinowski, 1923, p. 306), which means a contextual appropriation in which an utterance is located. Therefore, I removed the two less contextually dependent categories, “non-verbal audio” and “non-verbal visual”, to classify the examples into “verbal channel focused” and “visual channel focused” based on my case study Red and Rain. In addition, humour takes different forms across different platforms, in that users have actively participated online, and such participation helps redefine the process of meaning-making (Theo van Leeuwen, 2022). This will be explained and analysed later in the literature review and case analysis.

## Reviewing humour in the Chinese context of sharing videos

Subtitling humour became progressively more visible as a research subject (Chiaro, 2010; De et al., 2014; Wang, 2014; Bolanos-Garcia-Escribano, 2017). Nowadays, an increasing number of academics are interested in recreating meaning and communicating on Chinese social media platforms such as Weibo, WeChat, Tiktok, Momo, Bilibili, etc. (Wang and Feng, 2021; Wang and Crosthwaite, 2021; Chen et al., 2021; Chan, 2020). Few focus on the popular social media platform Little Red Book and the educational platform Rain Classroom, appealing to the younger generation with a particular focus on humour translation. This paper thereby aims to rectify that critical blind spot.

For contemporary Chinese audiences, translating humour using subtitles has shifted from the interplay between verbal and non-verbal modes (Yu, 2020) in the generation of humour to the decontextualising from the original and recontextualising the current Chinese social media context (Teng and Chan, 2022). The former refers to the interrelationship of verbal and non-verbal modes in understanding humorous film texts. The non-verbal channels include, in particular, paralanguage, kinesics, proxemics, cultural signs, sound effects and music (Ortega, 2011; Sala-Robert, 2016). The later shift indicates that in the new media era, in which video-sharing platforms are prevalent in the Chinese context, new possibilities are generated through modal shifts (i.e., from speech to writing and vice versa). In other words, an explanation of the short video has been decontextualised from the existing source texts and recontextualised on popular social video platforms to produce new and exciting video content or comments (Teng and Chan, 2022, p. 422). Humour occurs when such a process of recontexualisation in the Chinese social media contexts occurs. For instance, Wu and Fitzgerald (2021, p. 8) argue that “carnival creativity” can be found in Chinese social media when offering a bowl of bat soup to the developer of the teaching platform DingTalk. This is how online reviewers express their humour and sarcasm, referring to the perceived origin of the virus to the developer of the online teaching platform DingTalk that enables/forces learners to attend classes even though they are in the lockdown period COVID-19 Wu and Fitzgerald (2021, p. 4). This is a perfect case where bat soup is used to recontextualise the humorous imagery of the comment as a punishment for the developer that is not present in the original online teaching and learning context.

Martínex-Sierra’s (2006, pp. 290–292) classification of eight different types of humour in the dubbed version of the American sitcom *The Simpsons* can further explain the humour from Chinese social media platforms featured graphic, paralinguistic and non-marked elements of humour. I adapt his eight-humour classification (Martínex-Sierra, 2006, pp. 290–292) into three pertinent to the specificities of the two cases of my study:

1. Graphic Elements include the humour derived from a written message within an on-screen image.
2. Paralinguistic Elements include the non-verbal qualities of a voice, such as the intonation, rhythm, tone, timbre and resonance, which are associated with expressions of emotions.
3. Non-Marked (Humorous) Elements represent various instances that are not easily categorised as one of the other categories but are humorous.

The above classification can be understood as combining visual and non-verbal elements to interpret humour. Danmu comments are critical instances that help render humorous effects in Chinese social media. Posted across the video, it features “live” comments when viewers watch it (Yang, 2021, p. 1). This creates a sense of liveness and a co-viewing experience (Li, 2017; Ouyang and Zhao, 2016). Previous studies use diverse methods to examine humour in Chinese social media. Hsiao (2015) adopts monomodal pragmatic analysis to examine danmu comments that intend to evoke laughter with a particular reference to tucao. Zhang and Cassany (2019a, 2019b, 2019c) use multimodality to analyse the humorous effects drawn on an episode of a Spanish TV series *El Ministerio del Tiempo* (*The Ministry of Time*, in some places *The Department of Time*) (2015–2017, 2020–present) on Bilibili via danmu comments. Junru Mo (2022) argues that fan translation is an effective mediation to amplify the flow of humorous effects mainly through emoticons, bantering and spoofing. Although audiences as amateur translation users play an increasingly active role (Wu et al., 2018) in reshaping humour via danmu on video-sharing sites in China (Yang, 2021), less focus seems to favour the educational platform and short video themed with pedagogy. This paper attempts to contribute to this underexplored aspect by an in-depth analysis of collaborative user translation practice on how humour is rendered in Chinese social media Little Red Book and online teaching platforms Rain Classroom.

## Case analysis

The corpus selection includes two data sets drawn from the video-sharing platform Little Red Book and the online teaching platform Rain Classroom in the Chinese context. They are themed educational topics and embedded with new forms of humour. Termed by van Leeuwen (2006, p. 11), danmu comments that enabled by information technology are the “writing of the information age”. New writing presents “ideas through word and/or images, but achieves cohesion and coherence in their presentation increasingly through resources such as layout and colour schemes” (Djonov and Zhao, 2018, p. 11). This study aims to contribute to this topic but extends to the new type of humour embedded in social media platforms for contemporary Chinese audiences. As van Leeuwen (2008, p. 130) argues, a new emphasis should be placed on “the take-up of semiotic resources by users” because the analysis of the visual resources and their complexities, such as composition, movement colour and, gaining more visibility and importance compared to the analysis of images per se. Grouped under verbal and visual channel-focused humour, the new form of humour, as the case study will show, is not necessarily relevant to the source text but unique in the digital age.

### Data analysis

The first data set is collected from the video-sharing platform Little Red Book by the popular uploader Adam Chen陈老丝 (The Old Chen), who specialises in language learning and teaching. Adam runs his Little Red Book personal account while teaching at the International School of Ji Nan University. His followers reach 621, 000^1^ almost twice as of 20 January 2022 (319,000) in less than one and a half years. He divides his Little Red Book short video posts into two parts, humour posts and English learning posts. The selection of his uploaded short video belongs to the humour section, well-received, translation-related and hilarious, which is the focus—humour translation—on the social media platform for this study. Another set of data stems from the subjects of 30 postgraduate students who majored in translation and interpreting in my Multimedia Translation module. They are in their first year of the programme Master of Translation and Interpreting (MTI) in the 2022 autumn semester at the School of International Studies at Sun Yat-Sen University. The data shows danmu entries of teacher–student and student–student interactions throughout the course. The latter data set is selected because of the critical features of humour and the reflections of students’ voices that facilitate teaching and learning.

### Subtitling Little Red Book

Dissimilar to social media platforms outside of China, Chinese social media acts as “a distinctive form of mediated communication and practice” (Fitzgerald et al., 2022, p. 1), reflecting the unique interplay of technological changes driven by user-generated interactional practices such as tucao and danmu. Specifically, Little Red Book has been framed as a high-stake chance to push education to do more for pedagogical purposes. In Liang’s (2023, pp. 276–277) study, she positively correlated edited student-tailored clips with student participation, where clips driven by voice recognition technology, transcreated subtitles and interdisciplinary natures, such as reception studies, the creative economy and artificial intelligence could jointly use to optimise the viewing experience.

With a humour translation focus, the following examples group the humour drawn from Chinese social media platforms elastically under the verbal and visual channels. The user-generated additions are considered to make the humour accessible to viewers. They are enabled by danmu subtitling, transcreated subtitles and semiotic technology. Furthermore, to verify how humour appeals to audiences, the specificities and complexities of humour are also taken into account: swearing, bad jokes, vernacularism, non-contextuality and in-class humour.

**Example 1: Subtitling swearing through the user-generated humour enabled by danmu subtitling technology (verbal channel focused)**

**Fig. 1:**
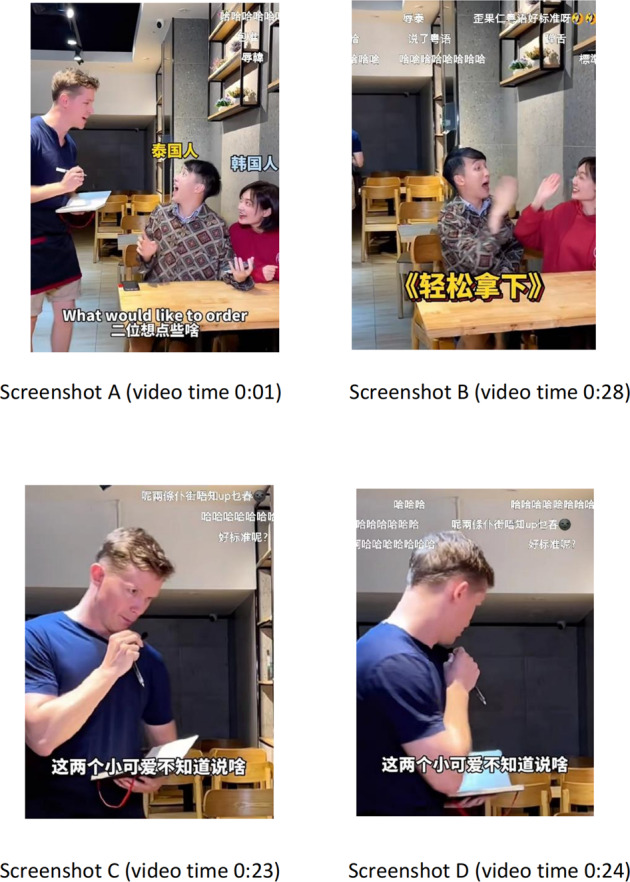
Screenshots for subtitling swearing of the data sample in Table 1. The figure indicates how swearing is subtitled and becomes an endearment in the situation of ordering food and drink.

This example^2^ tells that Adam and his friend Erna are ordering food and drink from a foreign server at a restaurant, as Screenshots A and B of Fig. 1 show. It is rather absurd and comical because they are labelled with the large, prominent Chinese characters “韩国人” (Korean) in yellow and “泰国人” (Thai) in blue, respectively, before they use these corresponding, strong accents and wear overreacted facial expressions prior to their actual ordering.

**Table 1:** The data sample for the video featured swearing.

Cantonese source text	Chinese translation	Danmu
呢两條仆街唔知 up 乜春 [I have no idea what these two poor guys said on earth.] (All Cantonese, Mandarin Chinese and danmu texts in the analysis section are offered back translation into English).	这两个小可爱不知道说啥 [I don’t know what these two little cuties said.]	呢两條扑街唔知 up 乜春 [I have no idea what these two poor guys said on earth.]

Humour comes in when they use the ridiculously heavy accent of Thai and Korean to make the order. However, the surprising mismatch and immediate humour of this example appear when the foreign waiter fluently and confidently utters an idiomatic Cantonese expression “呢两條扑街唔知up乜春” right after they make the order. As shown in Screenshots C and D of Fig. 1, the waiter complains Adam and Erna’s heavy accents (Thai and Korean English) by swearing, “呢两條扑街唔知up乜春” (I have no idea what these two poor guys said on earth.) with the inequivalent Chinese “这两个小可爱不知道说啥”. (I do not know what these two little cuties said.) Though there is a contrasting translationational adaptation from the swearing “扑街” (poor guys) to the endearment “小可爱” (little cuties) in the Chinese subtitles, the danmu subtitles provided by viewers compensate for the translation loss “呢两條扑街唔知up乜春  ”. (I have no idea as to what on earth these two poor guys said .)” added by an emoticon . This all-black face implies the waiter’s speechlessness, impatience and contempt about what exactly these two customers order.

Apart from the translation loss of the verbal channel, translation gain in the visual channel can be found thanks to the comparison between the waiter’s attitude with which he utters the swearing and the exciting reaction from both customers. He first looks around and then looks down at the talkie–talkie without looking at both customers, followed by a swift turnaround and a quick leave. His movement reveals that he swears while being afraid to be found out. On the contrary, Adam and Erna clap hands, showing their excitement because of their successful disguise of acting as local Thai and Korean, using heavy accents to order food and drinks. As Screenshot B illustrates, it is even more laughable when a super large label “轻松拿下“ (We nail him down!) pops up to show the easiness and confidence of such a disguise. Social semiotics is used in this instance to explain how taboo language and humour are vividly rendered through the all-black, speechless, awkward face emoticon and the ironic adaptation when “扑街” (poor guys) become “小可爱” (little cuties). The danmu comments in Fig. 1 clearly show that audiences can well recognise such humour and deliberate translation as they repeatedly respond “哈哈哈哈“ (hahaha). The term “semiotic resource” is a key term in social semiotics, referring to “a resource for making meanings” (Halliday, 1978, p. 192). Van Leeuwen (2005, p. 3) extends and redefines semiotic resources as

The actions and artefacts we use to communicate, whether they are produced physiologically—with our vocal apparatus, with the muscles we use to create facial expressions and gestures, etc.—or through technologies—with pen, ink and paper; with computer hardware and software; with fabrics, scissors and sewing machines, etc.

**Fig. 2:**
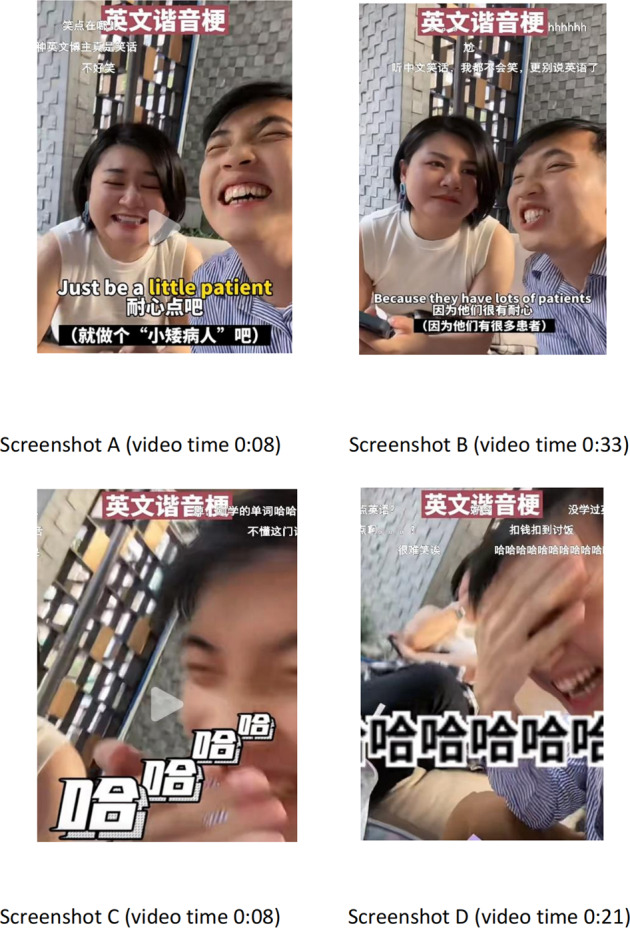
Screenshots for subtitling wordplay of the data sample in Table 2. The figure underlines how wordplay is creatively subtitled and maintained in the translation of bad jokes.

In this instance, vocal and visual apparatus is employed to interpret humour thanks to the waiter’s contempt facial expressions and run-away gestures, customers’ intended disguise and surprise, emoticon, and danmu technology where audiences act as a translator, consumer, questioner and answerer to optimise viewing experience when it comes to humour and humour-related translation. It is interesting to note that audiences even correct the spelling mistakes provided by the previous danmu comments “喺仆呀 唔喺扑” (It should be used the noun “仆” instead of the verb “扑”), question the pronunciation of the foreign waiter when he speaks fluent Cantonese “好标准呢?” (Is his Cantonese idiomatic?) affirmatively answered by another danmuer “標凖” (Idiomatic!), and criticise the way Erna uses exaggerated accents and labelled herself as Korean people “辱韩” (insulting Korean people). The semiotic resources surprisingly bring about knowledge-based user participation (Yang, 2021, p. 9) in multiple ways, such as checking the mistakes, evaluating the scene and criticising the topic’s significance. Swearing tends to be moderated from English into Chinese (Liang, 2018, pp. 35–37) and to be disappeared from Italian into English (Magazzù, 2018, p. 121), but what this case reveals is that the swearing is altered into endearment from Cantonese into Mandarin Chinese and is strengthened in the danmu subtitles supported by a funny face emoticon.

**Example 2: Subtitling wordplay through the user-generated humour enabled by transcreated subtitles (verbal channel focused)**

This instance only attracts some audiences if humour is favourably received among viewers thanks to danmu and subtitles in the previous example. Transcreated subtitles may compensate for such humour loss. As Liang (2023) put forward, transcreated subtitles are:

A new type of subtitling practice is a process by which fans and official bodies creatively translate and subtitle various types of audiovisual materials released in short-video and social platforms mainly for commercial, educational, self-entertaining and global sharing purposes, more often interlingually, intralingual and semiotically.

In this case,^3^, the English word “patient” as a homonym is used to make a humorous effect, as Table 2 illustrates. A doctor humorously and cleverly responds, “Just be a little patient”, to Adam’s mischievous and playful question, “Can I still grow a little taller?”. The wordplay applies to another bad joke justifying why doctors are so calm because they have many patients (patience).

**Table 2:** Data sample for the video featured with wordplay.

English source text	Chinese translation	Back translation
Adam: I asked my doctor, “Can I still grow a little taller”?	我问大夫, “我还能再长高点么? ”	I asked the doctor, “Can I still grow a little taller?”
My doctor said: Just be a little patient.	大夫说, ……(就做个“小矮病人”吧)。	The doctor said, ……Well, let’s be a short patient.
Adam: Why are doctors so calm?	为什么都那么镇定啊?	Why is so calm?
Because they have a lot of patience/patients.	因为他们很有耐心 (因为他们有很多患者) 。	Because they are very patient (because they have many patients).

The table indicates the English source text, Chinese translation and its back translation regarding Example 2.

However, discontent arises from the viewers showing that 10 out of 26 danmu dislike the bad jokes created by Adam in that these jokes are too deliberately presented to be fully appreciated, as shown in Table 3. It is important to note that the explanation of the homonym “patient” made by the viewers in both the danmu interface and the bottom commenting areas, on the one hand, facilitates viewers to understand the implying humour better; on the other hand, dilutes the immediate humorous reaction. The transcreated subtitles, including the exaggeratingly prominent Chinese characters “哈哈哈哈“ (hahaha) that replicate the mise en scène laughter, suggest the funniness of the jokes. Adam and Sophia’s hilarious facial expressions are funnier than the bad jokes per se. Plus, the specific colour of the yellow underling “a little patient” in Screenshot A and the foregrounding, escalating and flying subtitles “哈哈哈哈” (hahaha) in white in Screenshots C and D would be signs of humour. Furthermore, though the jokes are not particularly funny, the way Adam and Sophia talk, pause and interact could also be humorously suggestive. The seemingly non-semiotic behaviours are part and parcel of semiotic resources to the meaning-making process (van Leeuwen, 2005, p. 4).

**Table 3:** Data sample for some of the danmu entries that dislike Adam’s bad jokes in Fig. 2 and their back translations.

Danmu entries	Back translation
笑点在哪儿	Where is the laughing point?
不好笑	It is not funny.
尬	Embarrassing
听中文笑话, 我都不会笑, 更别说英语了	The Chinese jokes do not entertain me, let alone the English ones.
很难笑唉	It’s hard to get the funny part!

**Example 3: Subtitling orality through the user-generated humour enabled by semiotic resources (visual channel focused)**

**Fig. 3:**
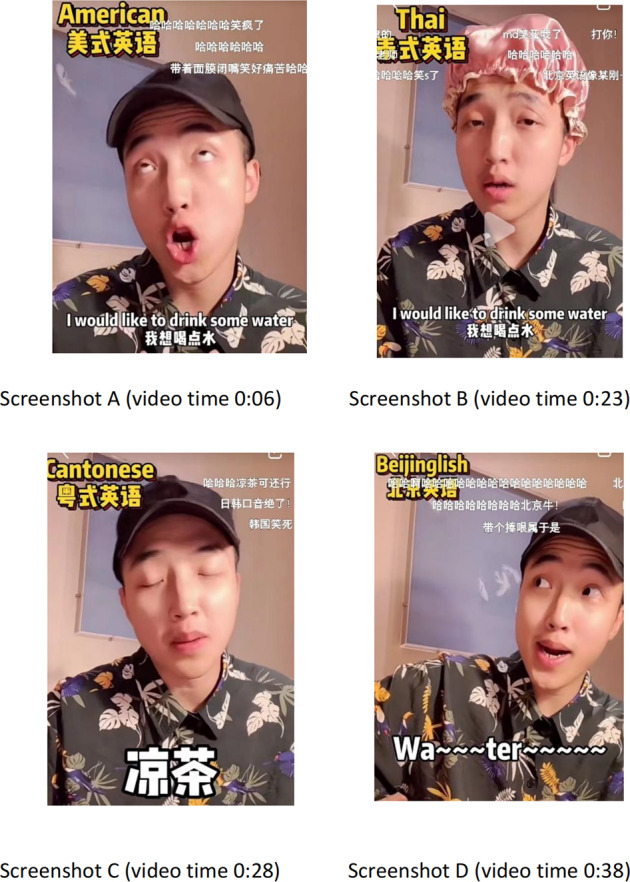
Screenshots for subtitling orality featured vernacularism of the data sample in Table 4. The figure shows how orality featured vernacularism is transcreated subtitled across eight different languages, dialects and cultures.

This instance^4^ indicates that Adam speaks the same line, “I would like to drink some water”, in eight different languages and dialects, British, American, Indian, Japanese, Korean, Thai, Cantonese, and Beijinglish. This short video is one of the most liked videos in the sub-channel of humour in Adam’s Little Red Book personal channel. Humour comes in when Adam humorously performs how these languages and dialects are spoken across different languages and cultures, which classifies this subtitling orality example into a visual channel. Four cases are particularly humorous because various semiotic resources are used to create the entertaining effect. As Screenshots A–D in Fig. 3 illustrates, they are American, Thai, Cantonese, and Beijinglish. Unlike adopting the universal strategy of simplification at the expense of idiosyncrasy of language style in the dubbing and subtitling contexts (Yang, 2022, p. 171), the Cantonese and Beijinglish cases, in this instance, employ adaptation and addition to transfer humour. The original “I would like to drink some water” has been altered to “I would like to drink some 凉茶”/Chinese herbal tea in the Cantonese version, the signature drink in Canton. However, it becomes a Beijing opera performance in the Beijinglish counterpart. The intonation and imagery of speaking the local languages have been well maintained that align viewers with the humorous connotation. The Thai case is even more creative and humorous because Adam wears a shower cap to imitate how Thai people causally talk, habitually wave hands, and frequently shake heads. The American case is one of the most humorous in this short video, possibly due to two reasons. First relates to its order of appearance, which comes right after the British accent. A sharp contrast occurs between the elegant and standard British pronunciation and Adam’s hoity-toity and exaggeratedly ridiculous version of American pronunciation. Second, the rhoticity in American English is emphasised (Costa and Serra, 2022) in this case as Adam exaggerates the pronunciation of the consonant [r] in an “r” position in the line “I would like to have some water”.

This instance informs a social semiotic approach with a focus on the multimodal features of humour. This humour is embedded in orality across different languages, dialects and cultures in the social media Little Red Book context drawn on Adam’s channel. The hat-wearing is associated with beauty in Thai women (Thepboriruk, 2019, p. 250). A funny shower hat-wearing could be an exaggerated sign of Thai people that is potentially hilarious in combination with Adam’s playful and comical facial expression. In this example, social semiotics is used to elicit humour when Adam’s overreacted gesture, mischievous gaze and passionate body position and posture in imitating different dialects and languages across cultures, which compounds a strong interaction between artefacts (the same line) and interaction (pitch, tone, gesture, gaze, posture) for gaining insight into meaning-making (Jewitt et al., 2016, p. 65). The humourous effect can also be supported by the wealth of the running danmu comments, some of which are listed in Table 4.

**Table 4:** Data sample for the video featured similarly humours expressions.

	American/Thai	Cantonese	Beijinglish
Source text	I would like to drink some water.	凉茶	Wa ~ ~~ter ~ ~~~~
Chinese/English Translation:	我想喝点水	Chinese herbal tea	Water (sung in Beijing Opera style)
Back translation from Chinese into English	I want to drink some water.	N/A	N/A

Three danmu entries that support the above similarly humorous expressions:

(1) 哈哈哈哈哈哈哈笑疯了[Hahahahahahaha, it’s crazily hilarious!].

(2) 日韩口音绝了 [The Japanese and Korean accents are marvellous!].

(3) 哈哈哈哈哈哈哈哈北京牛[Hahahahahahahaha! How great the Beijinglish accent is!].

### Subtitling the Rain Classroom

If subtitling the humour from Little Red Book is mostly situational-based and highly interactive, so does the Rain Classroom. Having Received popularity and success in learning and teaching, the educational platform Rain Classroom characterises “learner–learner, learner–instructor, and learner–content interactions”, assisting student initiative (Yu and Yu, 2022, p. 1217). A previous study has confirmed the effectiveness of blending Rain Classroom and Massive Open Online Courses (MOOCs) in a formal teaching context (Wang and Chen, 2018). Our study lies in applying the real-time danmu comments system into the practice of teaching and learning Multimedia Translation postgraduate courses, in which the above three-tier in-class interaction is visually and effectively achieved via danmu technology. Our course combines Tencent Meeting, the Chinese equivalent of Zoom in the West, and Rain Classroom to optimise online teaching and e-learning. Unexpected humour occurs as the following three instances enhance students’ immersion while learning remotely.

**Example 4: Subtitling in-class humour through the technology-driven humour by unstable internet connection (visual channel focused)**

In this case, danmu interface intends to facilitate teaching. Surprisingly, humour comes in when special visual effects occur in the danmu comments. One student raises a question via danmu of “为什么还有镭射效果” (Why do laser effects take place here?) in Screenshot A. That coincides with the teacher’s uncertainty if students are paid for an advanced member that allows such personalised and customisable settings for danmu appearance. Especially when this session is held before Halloween that may add to the touch of fear through the awesome animation effects.

Shortly, as Screenshot B indicates, a student replies to the raised doubt, “卡出阴影了” (This is simply because the weak internet connection leads to the shadowing effect). The class bursts into laughter, which can be proved in both the danmu comments and the comments left in the Rain Classroom in-class chat box. A sense of community is also built up when a relaxing atmosphere is created. In this way, it enables viewers to express their feelings, developing a sense of connectivity (Lee, 2021, p. 767).

**Fig. 4:**
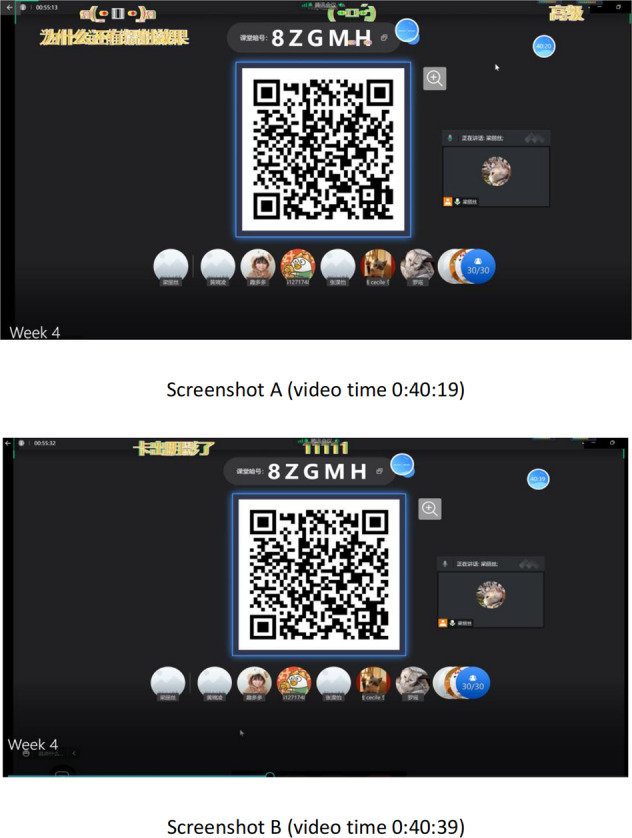
Screenshots for subtitling in-class humour of the data sample in Rain Classroom. The figure suggests how the unexpected in-class humour is captured and subtitled via the teaching platform Rain Classroom.

Humour arises in these polysystematic environments and social semiotic interactions because the weak internet connections enable the blistering effects of the danmu appearance that attracts mutual attention from the class. In this case, the signs refer to the technological advancement of the automatic shining effects, the uncertain discussion in the class and the bonding among the virtual sense of community. Multimodal modes consider all the contributions to the sign-making process (Kress, 2009, p. 54), as Fig. 4 indicates.

**Example 5: Subtitling the exercise-led humour enabled by sight translation (verbal channel focused)**

The humour in the class stems from the situation when the Danmu Etiquettes attached to Bilibili.com was first introduced to the class. The students were required to interpret the contents in English simultaneously. This sight translation task is challenging because simultaneous interpreting is often done independently in a given period. One student cleverly responds to my question, “Do we have any volunteers to complete this exercise?” as “我们决定一起上” (We all decided to interpret together). That makes the real-time exercise less demanding and lonely, although simultaneous interpreting/sight translation is not completed in such a teamwork manner^5^.

**Fig. 5:**
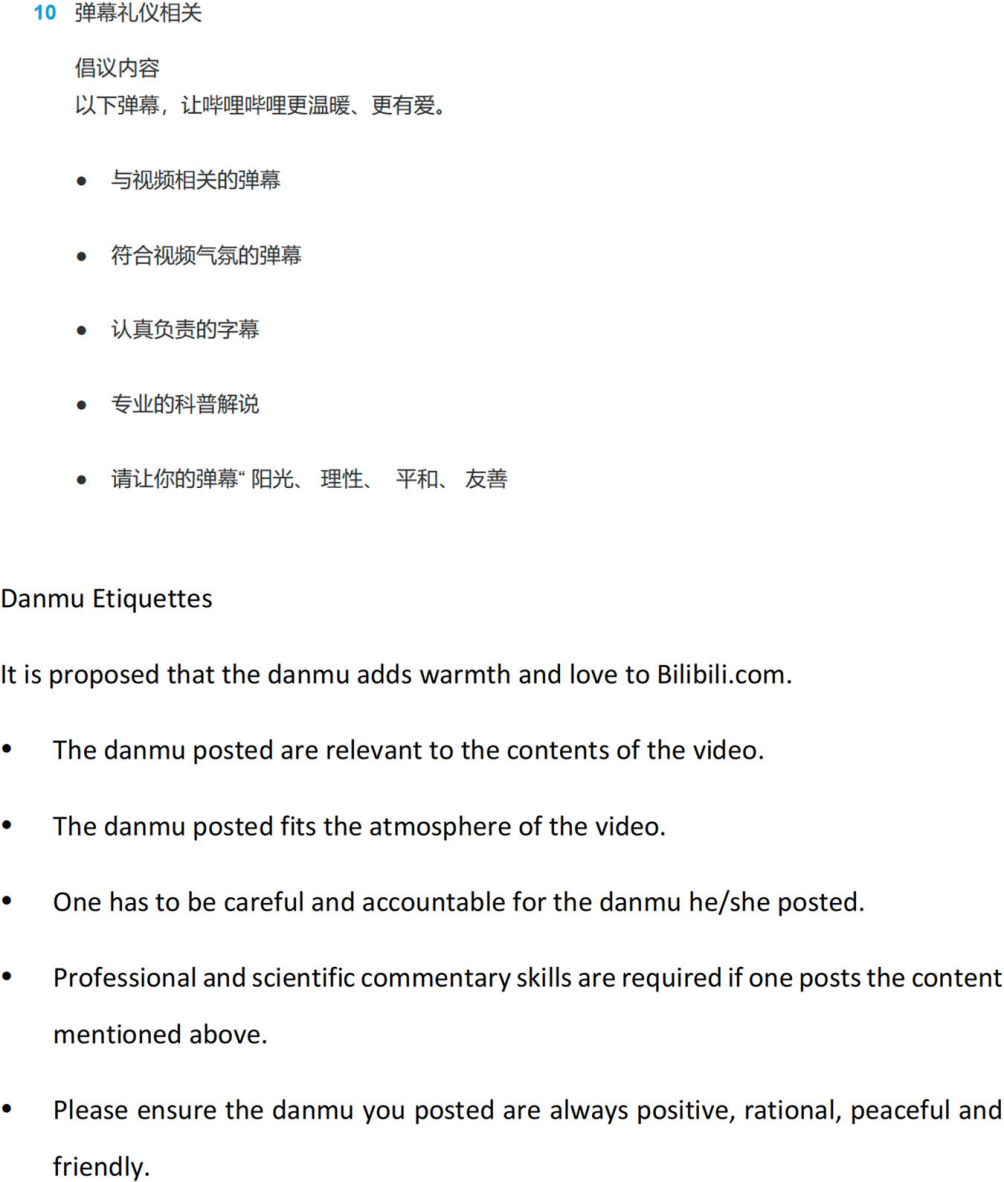
Screenshot for part of the Danmu Etiquettes extracted from Bilibili.com and their translation. The figure illustrates part of the Danmu Etiquettes taken from Bilibili.com, followed by their English translation.

The students’ sight translation turned out to need to be completed and mature compared to the standard Chinese translation I provided. One student posted one danmu entry criticising the above relay of interpretation as “extremely literal”, which is humorous because the exaggerated tone “extreme” and evident unsatisfaction “literal” is used. The humorous effect may stem from the sharp contrast between students’ super positive determination to complete the task as a team and the less satisfactory quality of their translation, verified by other students’ straightforward, impartial comments (Fig. 5 and Table 5).

**Table 5:** Data sample for some of the danmu entries extracted from the Multimedia Translation course that brings humour and their back translations.

Danmu entries	Back translation
我们决定一起上	We all decided to interpret together
The following danmu should make Bilibili warmer and lovely	N/A
Danmu should be relevant to the vibes of the video	N/A
Danmu should be responsible and serious	N/A
Professional scientific commentation	N/A
Please make sure your bullet screens are positive	N/A
May your Danmu kind and rational!	N/A
Extremely literal	N/A

**Example 6: Subtitling the exercised-led humour enabled by subtitling practice (verbal channel focused)**

If the previous example targets how humour travels through student commentation, the following instance extends our analysis of literal translation that may bring humorous effects. What Example 5 reveals is that the in-class humour shows up when the exercise is flexibly completed and how the students straightforwardly comment on their peer’s immature and unprepared work when the impromptu question is asked. On the contrary, this current example records a well-prepared subtitling homework that is presented in the class via danmu. We collected ten subtitled danmu entries of the dialogue extracted from the film *Jurassic World* (2015), three of which use literal translation when “more teeth” becomes the mere literal version, “牙齿更多” (more teeth). Though five of them adopt a less literal translational approach, “利齿更多” (more sharper teeth), linguistic incongruity still appears. As Chiaro (2010, p. 1) argues, verbal humour involves “matching the linguistic ambiguity in the source language with similar ambiguity in the target language”. However, when “more teeth” is literally transferred to target audiences, which may imply “更凶狠、凶猛” (in danmu translations (3), (4) and (8)) (fiercer, more violent). Such implication, colloquialism and rhetoric (using teeth to refer to fierceness and violence, e.g., in danmu translation (8)) are better conveyed to target audiences rather than merely using literal translation (more teeth). Eight out of ten translations directly maintain the imagery of “teeth”, which makes the situation ridiculous because students are unaware of the source text’s intention but carelessly post the direct translation via danmu. It is surprising to discover that students are more willing to express themselves through danmu technology online partly because the response and reaction (laughter in this case) from others are not as prompt as that in the offline class. This is because once the teacher points out how nonsensical and silly to repeat the “more teeth” version on the spot, students will realise the comical translations they provided (Table 6).

**Table 6:** Data sample for some of the students’ Chinese subtitling work of *Jurassic World* (2015) and their back translations.

Source text	Chinese translation by students	Back translation
(Our DNA excavators discover new species every year.) But consumers want them bigger, louder, and more teeth.	1) 然而, 游客们希望恐龙体型更大, 叫声更响, 牙齿更多。	However, visitors hope dinosaurs have bigger figures, louder shouts and more teeth.
	2) 顾客们希望他们体格更大, 叫声更响, 牙齿更多	Consumers hope they have a bigger figure, shout louder and have more teeth.
	3) 游客想看到体积更大 吼叫声更响 更凶狠的恐龙	Visitors want to see dinosaurs that are bigger, louder and fiercer.
	4) 更大、更凶猛, 牙齿更锋利	Bigger, fierce, sharper teeth.
	5) 身型更高大、叫声更威猛、利齿更多	Taller, bigger figure, louder, more powerful shout and sharper teeth.
	6) 但是客户希望恐龙更有震慑力, 够大、够狠、牙齿够多	But clients hope dinosaurs are more powerful, bigger, more violent and have more teeth.
	7) 但游客希望恐龙更大, 叫声更响, 利齿更密	But visitors hope dinosaurs are bigger, louder and have more teeth.
	8) 更大, 更猛, 更狠	Bigger, fiercer, more violent.

## Conclusion

Expanding Little Red Book’s imagery beyond a set stereotype formerly known as practical travel and product reviews (Fitzgerald et al., 2022) and a female-led form of user expression and content consumption (Chen and Liu, 2023), our study suggests that it also functions as an effective vehicle to transfer humour in new and exciting ways. Thanks to danmu technology and transcreated subtitles, humour is innovatively adapted in the digital mediascape to cater to its younger generation that virtually established a gradual sense of community. To share common ground on embedding humour translation, Rain Classroom is blended into our study to explore further how humour is surprisingly created through a multitude of semiotic possibilities. Not confined to “performance expectancy, effort expectancy, social influence, facilitating conditions, and attitude” (Yu and Yi, 2020, p. 79) in linguistics classes, our study proves that Rain Classroom excel in Audiovisual Translation-related course that encourages students’ participation and peers evaluation associated with sight translation, simultaneous interpreting, dubbing and subtitling exercises that bring insights throughout students’ before-in-and-post class reflections. Humour plays a pivotal role in inspiring students in the process of consuming, creating and engaging in the social media and educational platform Red and Rain.

The concluding remarks are also revolved around the answers to the below three research questions set at the beginning of this paper.

1. What characterises humour in the Chinese social media Little Red Book and online teaching platform Rain Classroom?
  As to the first research question regarding the humorous features of the case-study social platform Little Red Book and the online teaching platform Rain Classroom, the results are inevitably subjective rather than objective because limited examples have been extracted from them. However, based on the selected, popular and representative instances instead of the exhaustive numbers of examples, these social platforms, characterised by education and pedagogical natures, have the following telling features regarding humour: light swearing, wordplay-led, orality-oriented, and in-class surprisingly unexpected. The unexpected humour in the course relates explicitly to technical issues, impromptu questions, students’ freedom of choosing the way of answering questions, harsh style of students’ evaluation and lack of criticism.
2. How to translate such humour in the Chinese social media Little Red Book and online teaching platform Rain Classroom?
  The translation is achieved through collaborative efforts made by viewers who successively and accumulatively contribute danmu, transcreated subtitles and chat-box comments. That enables humorous effect in the typical and specific new type of user-generated and semiotic-enabled humour in subtitling swearing, wordplay, orality and in-class unexpected situations. Though translation loss is in sight at some point, translation gain is creatively reached thanks to the social semiotic multimodal approach. Furthermore, a potential sense of community and connectivity is also established due to the mutual work of translation, manipulating ways of addressing viewers’ different languages, cultural identities, and self-expression (Lee, 2021, p. 767).
3. What potential practical, methodological and theoretical contributions does humour translation bring to translation studies?

When it comes to the ultimate contribution to translation studies, practically, humour does travel across different social platforms, languages and cultures because of the blurring boundaries between professional and non-professional translation, mainly because of the increasingly important role played by the users that enable a higher and more creative audience participation and user engagement in translation studies. Methodologically, a new type of humour is generated in five sub-categories in visual and verbal channels drawn from social media and online teaching platforms in the Chinese context. They are verbal channel-focused, user-generated swearing humour, verbal channel-focused, user-generated wordplay humour, visual channel-focused, user-generated orality humour, visual channel-focused, technology-driven in-class humour, and verbal channel-focused user-generated in-class humour.

Last but not least, theoretically, this paper contributes to translation studies taking social semiotics and polysystem theory to explore how the above new type of humour is translated, adapted and recreated in Chinese social media and online teaching contexts. Such a new type of humour translation is inaugurated by the unprecedented growth of audiences and readers as internet users and translators in the digital age that initiates and carries out translation-related online activities and builds a sense of community and comradeship.

This study is not without limitations. Apart from short-video sharing and educational platforms, future work should consider more thematically heterogeneous social media platforms to expand the scope of the study as to how humour travels in the digital mediascape. Moreover, our study focuses on Chinese social media platforms and their translation from Chinese into English. It would increase our understanding of the relatively recent practice that sustains this digital humour of the reversal of established global translation flows from foreign languages into Chinese. Finally, further research relating to geographically diverse national and cultural identities ranging from the transfer of humour to ideology, gender, sexuality, etc., drawn on social media platforms, would be worthwhile in its own right and for its potential to make insightful comparisons across a variety of contexts.

## Data Availability

The datasets generated during and/or analysed during the current study are available from the corresponding author upon reasonable request.

## Footnotes

1 The data are updated on 14 April 2023, available from https://www.xiaohongshu.com/user/profile/5b66db6a6b58b77ec5697a48. Accessed 31 May 2023.

2 The example is extracted from Adam’s Little Red Book channel https://www.xiaohongshu.com/discovery/item/62c13b340000000006039607?app_platform=android&app_version=7.60.0&share_from_user_hidden=true&type=video&xhsshare=WeixinSession&appuid=608144590000000001006394&apptime=1666717435. Accessed 31 May 2023. The web link is without the danmu interface, please consult the application Little Red Book for a fuller post containing the danmu information and interface. There is a similar version released on Erna’s Little Red Book channel https://www.xiaohongshu.com/discovery/item/62f1db2300000000140298dd?app_platform=android&app_version=7.60.0&share_from_user_hidden=true&type=video&xhsshare=WeixinSession&appuid=608144590000000001006394&apptime=1666757088. Accessed 31 May 2023, but Adam and Erna order different drinks. This one is not used for analysis because it does not contain any danmu comments from viewers.

3 This example stems from Adam’s Little Red Book personal channel https://www.xiaohongshu.com/discovery/item/62863f86000000000102bf5d?app_platform=android&app_version=7.60.0&share_from_user_hidden=true&type=video&xhsshare=WeixinSession&appuid=608144590000000001006394&apptime=1666840464. Accessed 31 May 2023.

4 This example is available from https://www.xiaohongshu.com/discovery/item/5ee64995000000000101d74c?app_platform=android&app_version=7.60.0&share_from_user_hidden=true&type=video&xhsshare=WeixinSession&appuid=608144590000000001006394&apptime=1666933752. Accessed 31 May 2023.

5 Admittedly, simultaneous interpreting is a cognitive demanding task where two interpreters rotate every 30 min in a booth to overcome fatigue and cooperate (Chmiel, 2008, p. 261). It is a teamwork-based task, but our paper discusses the short-term simultaneous interpreting/sight translation that is far less demanding and expected to be completed independently.
